# Coal Miners and Lung Cancer: Can Mortality Studies Offer a Perspective on Rat Inhalation Studies of Poorly Soluble Low Toxicity Particles?

**DOI:** 10.3389/fpubh.2022.907157

**Published:** 2022-07-15

**Authors:** Robert J. McCunney, Mei Yong

**Affiliations:** ^1^Harvard Medical School, Brigham and Women's Hospital, Boston, MA, United States; ^2^MY EpiConsulting, Duesseldorf, Germany

**Keywords:** coal miners, lung cancer, PSLTs, poorly soluble particles, carbon black, titanium dioxide

## Abstract

Inhalation studies involving laboratory rats exposed to poorly soluble particles (PSLTs), such as carbon black and titanium dioxide, among others, have led to the development of lung cancer in conditions characterized as lung overload. Lung overload has been described as a physiological state in which pulmonary clearance is impaired, particles are not effectively removed from the lungs and chronic inflammation develops, ultimately leading to tumor growth. Since lung tumors have not occurred under similar states of lung overload in other laboratory animal species, such as mice, hamsters and guinea pigs, the relevance of the rat as a model for human risk assessment has presented regulatory challenges. It has been suggested that coal workers' pneumoconiosis may reflect a human example of apparent “lung overload” of poorly soluble particles. In turn, studies of risk of lung cancer in coal miners may offer a valuable perspective for understanding the significance of rat inhalation studies of PSLTs on humans. This report addresses whether coal can be considered a PSLT based on its composition in contrast to carbon black and titanium dioxide. We also review cohort mortality studies and case-control studies of coal workers. We conclude that coal differs substantially from carbon black and titanium dioxide in its structure and composition. Carbon black, a manufactured product, is virtually pure carbon (upwards of 98%); TiO_2_ is also a manufactured product. Coal contains carcinogens such as crystalline silica, beryllium, cadmium and iron, among others; in addition, coal mining activities tend to occur in the presence of operating machinery in which diesel exhaust particles, a Type I Human carcinogen, may be present in the occupational environment. As a result of its composition and the environment in which coal mining occurs, it is scientifically inappropriate to consider coal a PSLT. Despite coal not being similar to carbon black or TiO_2_, through the use of a *weight of evidence* approach-considered the preferred method when evaluating disparate studies to assess risk- studies of coal-mine workers do not indicate a consistent increase in lung cancer risk. Slight elevations in SMR cannot lead to a reliable conclusion about an increased risk due to limitations in exposure assessment and control of inherent biases in case-control studies, most notably confounding and recall bias. In conclusion, the weight of the scientific literature suggests that coal mine dust is not a PSLT, and it does not increase lung cancer risk.

## Introduction

Inhalation studies involving laboratory rats exposed to poorly soluble particles (PSLTs), such as carbon black and titanium dioxide, among others, have led to the development of lung cancer in conditions characterized as lung overload. Lung overload has been described as a physiological state in which pulmonary clearance is impaired, particles are not effectively removed from the lungs and chronic inflammation develops, ultimately leading to tumor growth. Since lung tumors have not occurred under similar states of lung overload in other laboratory animal species, such as mice, hamsters and guinea pigs, the relevance of the rat as a model for human risk assessment has presented regulatory challenges.

Recently, an international panel of scientific and regulatory toxicology, epidemiology and particles scientists discussed the relevance of rat lung tumor data for poorly soluble low toxicity particles (PSLTs) (1). Their consensus views were: “*In summary, the Expert Panel thoughtfully considered the current state of the science for PSLT and reached agreement on several matters relevant to PSLT toxicology, hazard classification and risk assessment*. Specifically, the Expert Panel: (1) outlined an experimental process for determining if a material should be considered as poorly soluble and low toxicity; (2) agreed the rat is a sensitive test species for PSLT inhalation toxicology and supported continued use of the rat for PSLT inhalation toxicology studies; (3) recommended that future studies focus on defining thresholds for inflammation and inflammation be used as a critical endpoint for OEL setting; (4) agreed rat lung cancer occurring only under conditions of lung particle overload, in the absence of corroborating data from other species, should not be interpreted to imply a cancer hazard for humans; and (5) were in consensus that rat lung tumors under lung particle overload are not relevant to health hazard or risk under non-overload exposure conditions.”

Inhalation studies of PSLTs, such as carbon black and titanium dioxide, among others, in which lung cancer under conditions of lung overload has occurred in rats, but not mice, hamsters or guinea pigs, has presented regulatory challenges in human risk assessment. It has been suggested by some that coal miners may represent an occupational group in which it may be argued that lung overload has occurred, primarily due to substantial amounts of retained coal dust in miners with coal workers pneumoconiosis, a chronic inflammatory condition that seems primarily due to the iron content in coal and not quartz (2).

It should be noted, however, that biomathematical models have not demonstrated physiological evidence of lung overload in humans (3). The authors studied 131 US coal miners with an average cumulative dust exposure of 107 mg-year/m^3^ with 36 years of exposure and a mean coal mine dust concentration of 3 mg/m^3^). In the biomathematical analysis, a mean dose of 13.8 grams of coal dust were retained in the miners studied,and found that a three-compartment model with no clearance break-down fit the lung burden best (3).

UK investigators also used statistical and mathematical modeling techniques to analyze data from an autopsy results of 423 UK miners to predict lung and lymph node dust burdens in coalminers with long-term exposure to respirable dust (4). The analysis was based on autopsy data held at the Institute of Occupational Medicine. The mean lung dust burden was 14.4 g (sd = 11.7 g) Like Kuempel et al. described above, Tran and colleagues showed that coalminers did not develop overload even under high exposure scenarios.

In summary, intensive investigations in the US and in the UK showed that coalminers did not develop overload–even under high exposure conditions and are not at risk of lung cancer (5).

Despite these mathematical analyses that indicate that coal miners do not develop “lung overload” as physiologically defined, the term “lung overload” is often used colloquially in regulatory and other settings to describe the extent of dust retention in coal miners in comparison to other occupational groups. In this discussion, we refer to coal miners with pneumoconiosis as representing a clinical state of “overload” as a result of substantial levels of retained dust in the lungs and the corresponding level of chronic inflammation.

As a result, it seems wise for the purpose of this review to raise the question that If coal miners do experience “lung overload” could studies of risk of lung cancer among coal miners provide a perspective on the human significance of rat inhalation studies of poorly soluble low toxicity particles? This question necessitates addressing not only whether coal increases risk of lung cancer but also whether coal is an appropriate surrogate for carbon black and other PSLTs?

The purpose of this report is to (1) address whether coal is a PSLT and an appropriate surrogate for carbon black and titanium dioxide (TiO_2_), as examples of PSLTs and (2) whether coal worker mortally studies show an increased risk of lung cancer (6).

## Methods

To address the questions posed about the relevance of coal as a surrogate for PSLTs and to evaluate coal worker mortality studies regarding lung cancer risk, we (1) reviewed the components of coal, carbon black and TiO_2_ and (2) identified and reviewed the published coal worker mortality studies.

### Addressing Compositional Differences Between Coal, Carbon Black and TiO2

The International Agency for Research on Cancer (IARC) convened a working group in 1997 to address potentail carcinogenic risks in coal miners (7). As in most IARC monographs, considerable attention is devoted to the physico-chemical aspects of the substance under review. IARC, considered an authoritative source, thoroughly reviewed numerous aspects of coal, including its composition. Althought the monogaraph is now over 20 years old, the basic compositional aspects of coal will not be substanatilly different.

### Literature Review of Coal Worker Mortality Studies

The same IARC working group described above that reviewed coal in 1997 also reviewed epidemiology mortality studies of coal workers. The results of these studies will be tabulated. In addition, to identify mortality studies of coal workers published after the 1997 IARC monograph, we conducted an updated literature review *via* PubMed.

## Results

### Composition of Coal

Is coal an appropriate surrogate to evaluate risk of lung cancer from exposure to poorly soluble particles such as carbon black and titanium dioxide?

Coal is a complex mixture of more than 50 elements and their oxides; the mineral content varies with particle size of the dust and the coal seam (7). The remaining portion consists of a variable mixed dust, introduced into the mine atmosphere through operations other than coal cutting, such as roof bolting or in the distribution of rock dust (a low-silica limestone dust) to prevent explosions. Airborne respirable dust in underground coal mines has been estimated to be 40–95% coal [(8); United States National Institute for Occupational Safety and health, 1995]

In addition to coal itself, coal mining has involved the use of heavy equipment, often run by diesel engines. Operation of diesel equipment underground can lead to the generation of fine dust particulates (< 1 μm); the composition of which would be fairly typical of diesel exhaust from industrial machines. Diesel exhaust particles (DEPs) are considered a Type I Human Carcinogen (9). The presence of DEPs in the coal mining environment presents a significant confounder in evaluating the significance of lung cancer results in coal worker mortality studies. Below are a series of tables that describe components of coal.

Table 1 notes the carbon content of various types of coal. As noted, the carbon content can vary from 50% in Peat to 92 to 95% in anthracite (7).

**Table 1:** Carbon contents of coal.

**Coal type**	**Rank**	**Carbon %**
Peat		50–65
Lignite	Low	65–70
Sub-bituminous	Low	75–80
Bituminous	Intermediate	80–90
Semi-bituminous	Intermediate	90–92
Anthracite	High	92–95

As noted in Table 2 below, coal also contains Type 1 Human Lung Carcinogens per the International Agency for Research on Cancer (IARC) classification system, including beryllium, cadmium and chromium. The presence of these substances presents significant confounders in evaluating any potentail lung cancer risk in coal miners

**Table 2:** Elements and trace elements in coal.

**Constituent**	**Range (Percentage)**	**Constituent**	**Range (ppm)**
Aluminum	0.43-3.04	Beryllium	0.2–4
Iron	0.34-5.32	Cadmium	0.1–65
Silicon	0.58-6.09	Chromium	4–54
Titanium	0.02-0.15	Nickel	3–80

Quartz is another component of coal that varies from about 4–8% in British Coal mines (see Table 3 below). In addition, quartz concentrations in US and German coal mines can vary up to 7 and 5%, respectively [see Tables 7, 8 from IARC's monograph on Coal (7)].

**Table 3:** Compositional data for airborne dusts in a sample of British coal mines prior to 1970.

**Coalfield**	**Quartz %**
Scottish	5.8
Cumberland	6.8
Yorkshire	7.8
North Wales	6.9
Warwick	4.2

^*^Abstracted from IARC (7); Table 6.

As noted in Table 4 below, coal contains concentrations of quartz ranging from 3.2 to nearly 7% in British coal mines.

**Table 4:** Quartz percentages in samples of coal dust: USA: 1985–1992.

**Occupation**	**Average quartz content**
Roof Bolter	6.97
Miner operator	5.54
Shuttle car operator	4.33
Longwall shearer operator	4.02
Coal Drill operator	3.29

*From Tomb et al. (11); abstracted from IARC (7); Table 7*.

Table 5 below notes the percentage of quartz content by weight and corresponding exposure levels in dust in German coal mines.

**Table 5:** Mean quartz content in dust in German coal mines.

**Particle size**	**Quartz (% by weight)**	**Dust concentrations (mg/m^**3**^)**
Total dust	4.1 ± 3.3	53.1 ± 29.4
Dust <7 microns	4.3 ± 3.0	25.3 ± 13.0
Dust <5 microns	2.9 ± 1.9	9.2 ± 7.9
Dust <3 microns	2.2 ± 1.6	2.1 ± 1.6

*From Leiteritz et al. (12); abstracted from Table 9 (7)*.

In summary, coal contains varying concentrations of carbon as well as carcinogens such as crystalline silica, beryllium and cadmium. In addition, coal mining often necessitates the use of diesel powered machinery that can lead to the generation of diesel exhaust particles, considered by IARC to be a human lung carcinogen.

### Carbon Black

Carbon black is an intentionally produced substance that is generated by the incomplete combustion of petroleum products, most notably heavy oils. Unlike the multi compositional aspect of coal, carbon black is virtually pure carbon.

According to an update on carbon black in Pattys' Industrial Hygiene and Toxicology text, “*Carbon black is the earliest known synthetic pigment, having been produced by the Chinese more than 1,500 years ago...Carbon black has been commercially produced in the United States for more than 100 years*. Its major use has been as a reinforcing agent in rubber, particularly in tires” (10). “In contrast to diamond and graphite, carbon black is an amorphous carbon composed of particles and fused particle aggregates. Untreated carbon black grades generally contain more than 97% elemental carbon with variable amounts of oxygen, hydrogen, and sulfur. Less than 1% of carbon black particles consist of extractable organic materials. The extractable material, usually in the range of tenths of 1% by weight of the carbon black, consists of a mixture of polycyclic aromatic hydrocarbons (PAHs), lesser amounts of other polynuclear aromatic hydrocarbons (PNAs), and sulfur compounds” (10).

Table 6 below demonstrates the significant compositional differences between carbon black and coal, most notably that carbon black is virtually pure carbon, whereas as noted earlier in this report, carbon content in coal varies widely and can be as low as 65% in lignite.

**Table 6:** Components of commercially produced carbon blacks^*^.

**Property**	**Acetylene black**	**Furnace black**
Carbon (%)	99.8	97.3–99.3
Hydrogen (%)	0.05–0.10	0.45–0.710
Oxygen (%)	0.10–0.15	0.19–1.25
Benzene-extractable organics (%)	0.1	0.01–0.18
Ash (%)	0.00	0.1–1.0
Sulfur (%)	0.02	0.05–1.5

^*^Abstracted from Table 89.3 of Carbon Black, Pattys Industrial Hygiene and Toxicology, 2012.

### Titanium Dioxide (TiO_2_)

Titanium dioxide is a white inorganic compound, which has been used for around 100 years in a vast number of diverse products for its non-toxic, non-reactive and luminous properties, which heighten the whiteness and brightness of many materials.

Titanium dioxide has reflective characteristics and is known for being the whitest and brightest of known pigments, with reflective qualities. This compound has reflective properties and can also both scatter and absorb UV rays. Applications include paints, paper, plastics, pharmaceuticals, sunscreen and cosmetics and skin care, as well as food.

Metallic titanium and TiO_2_ forms are insoluble, unreactive, non-metabolized, and virtually devoid of acute toxicity; although chronic overload inhalation exposures to high concentrations of TiO_2_ in rat studies can cause lung tumors in particle-exposed rats. With expansion of nanotechnology, the TiO_2_ material has been engineered in terms of a variety of shapes and sizes resulting in significant particle size reduction. The reduction of the particle size leads to increased specific surface area, which contributes to increased potential for toxicity of some forms of the engineered TiO_2_ nanomaterials.

### Toner Mortality Study

Copier toners are fine powders composed primarily of plastics and small quantities of colorants and functional additives. They appear to be fit a description of a poorly soluble low toxicity particle. To evaluate potentail carcinogenic risks from occupational exposure to copier toner, investigators conducted a retrospective mortality study of 33,671 workers potentially exposed to copier toner (28). This study showed statistically significant deficits in all cancers, including lung cancer. No increase in nonmalignant respiratory disease was shown.

### Coal Worker Mortality Studies

Despite the limitations of using coal as a surrogate for poorly soluble particles like carbon black and TiO_2_, let's briefly review the highlights of coal worker mortality studies.

Risk of lung cancer among coal miners has been investigated in cohort mortality studies conducted over nearly 50 years. Over 120,000 coal miners have been evaluated in UK, Germany, Netherlands, USA, Poland, Japan and Australia. Epidemiological studies provide data regarding the risk of lung cancer in workers exposed to coal dust. See Table 7 below that summarizes the key results of coal worker mortality studies.

**Table 7:** Summary of 15 cohort studies of coal miners with lung cancer standardized mortality ratios (SMR) and 95% confidence intervals (CI).

**Cohort studies**	**Countries**	***N***	**Lung Ca. SMR**	**95% CI**
Liddell (13)	UK	3,169	0.63	n.a.
Costello et al. (14)	US	3,726	0.67	0.4–1.0
Rockette (15)	US	23,232	1.13	1.0–1.3
Armstrong et al. (16)	AUS	213	0.25	0.01–1.4
Atuhaire et al. (17)	UK	3,865	0.78	0.7–0.9
Kuempel et al. (18)	US	8,878	0.77	0.6–1.0
Swaen et al. (19)	NLD	2,941	1.02	0.9–1.2
Starzynski et al. (20)	POL	7,065	1.07	0.9–1.2
Brown et al. (21)	UK	23,630	0.74	0.50–1.06
Miyazaki and Une (22)	JP	5,818	<15 yr: 1.00	0.41–2.43
			>15 yr: 2.08	1.01–4.27
Morfeld et al. (23)	DE	4,581	0.79	0.64–0.96
Attfield and Kuempel (24)	US	8,899	1.07	0.95–1.19
Miller and MacCalman (25)	UK	17,820	0.99	0.93–1.05
Graber et al. (26)	US	9,033	1.08	1.00–1.18
Tomášková et al. (27)	CZ	6,687 with no CWP	0.83	0.70–0.98
		3,476 with CWP	1.70	1.41–2.04

As noted in the table, the overwhelming evidence of the cohort mortality studies shows no significant increase in risk of lung cancer among coal miners. Standardized Mortality Ratios (SMRs) are typically <1 and when above 1, the results are usually not statistically significant.

There are two cohort studies (11 and 12) and a recent case-control study (29) that suggest a minor risk elevated risk in lung cancer. The risk estimates from the case-control studies are not in agreement with the results from the cohort studies. The inherent limitation of case-control studies, with lack of clear temporality, sampling bias and information bias is of limited value for a causality evaluation. Nevertheless, these studies will be described below.

In an updated study of US coal miners, Lung cancer SMR was slightly elevated and of marginal statistical significance- as the lower limit of the 95% confidence limits was 1 (SMR = 1.08, 95% CI: 1.00–1.18) (26). It is noteworthy that an earlier follow-up of the same cohort of upwards of 9,000 coal miners through 2,000, showed no association between coal mine dust exposure and lung cancer (24).

This excess in the updated US study has been described as “*unexceptionable”* (5). In this assessment, the authors stated: “*Internal analyses showed an association of lung cancer mortality with coalmine dust exposure but only during the last follow-up interval from 2000 to 2007”* (26).

The US study has a number of limitations that limit drawing broad conclusions about lung cancer risk among coal miners, most notably because the study relies on smoking information collected *only* at the start of follow-up; the models used are unable to adjust for smoking habits after leaving work. The coal miners at the start of the study (1969/1970) smoked cigarettes less than current smokers in the US male population (prevalence of smoking more than 25 cigarettes per day: 12.4% among US coalminers vs. 28.0% in the US male population). This difference has been attributed to prohibition of smoking when miners were working underground. As a result, it is not unreasonable to assume that smoking coal miners increased their intensity of smoking after cessation of coal mining work, and as a result, increased the lung cancer mortality rate of the cohort during the last follow up period when most coalminers had stopped working underground. This issue has also been discussed in the most recent study of UK coal miners, which is the largest study of coal miners to date with better assessment of exposures in a time-dependent manner (25).

The US study is further limited by an incomplete assessment of jobs held, including no start and end date of jobs held before 1969/1971; no information on jobs held after start of follow-up in 1979/1971 and no end date of working as coalminer for 16% of cohort members. Thus, only a crude assessment of exposure to coalmine dust up to the start of follow-up was possible and no time-dependent exposure analysis or lagging or lugging of exposures could be done. Measurements of crystalline silica, an acknowledged Group 1 IARC human carcinogen, were available only after 1982 but had to be allocated to the jobs held before 1969/ 1971. Recall that this updated US publication is a further analysis of US coal miners, in which a mortality study of upwards of 9000 coal miners showed no elevation in lung cancer (24).

Taeger et al., who later performed a case-control study, in a letter to the editor about the Graber study, noted that “emphasizing a ‘significant' relationship with lung cancer mortality seems inappropriate in view of borderline results, [as noted above, the lower limit of the 95% CI was 1.0] lacking exposure-response relationship and nonsignificant results of the categorical analysis. Second, SMRs between regions differ considerably, especially those for lung cancer—significantly up to a factor of 3. We would suggest using regional rates for each region and then combining results to avoid bias” (Taeger et al., 2014). Note the importance of using regional rates as a reference group.

A year after the US study, European investigators published a case-control study in which risk of lung cancer among coal miners was addressed (29). Described as the European Synergy Study, the authors evaluated the joint effect of smoking and occupational lung carcinogens in 14 case-control studies comprising 14,251 lung cancer cases and 17,267 controls. Exposure assessment was based on: Employment duration; Time since first employment; and Job titles maintained for at least a year. Exposure concentrations for coal dust or other lung carcinogens-were not available.

This case-control study examined lung cancer risk for coal and ore miners and quarrymen, using the European SYNERGY data base. SYNERGY investigated the joint effect of smoking and occupational lung carcinogens. Numerous studies have been published based on the SYNERGY data base. This study focused on one aspect of SYNERGY: coal miners and lung cancer risk. The quantitative exposure assessment was based on employment duration and time since first employment. For coal miners, employment duration of 1–9 years (OR = 1.46; 95% CI: 1.18–1.80) and ≥20 years (OR = 1.73; 95% CI: 1.14–2.62) implied increased risks, while employment duration of 10–19 years suggested no link with lung cancer (OR = 0.99; 95% CI: 0.67–1.47). This latter pattern is inconsistent with a dose response relationship between coal mining exposure and lung cancer risk. Assessing dose-response is a critical step in evaluating potential causality.

Detailed information on smoking does not compensate for the weakness of the occupational exposure assessment. Exposure to coal mine dust was based on job titles maintained for at least a year. No exposure concentrations- for coal dust or any other known lung carcinogens-were available. As a result, confounding effects from lung carcinogens such as crystalline silica, asbestos, PAH, radon and metals could not be addressed in the analysis. Therefore, the observed association cannot be directly attributed to coal dust. Employment duration was used as proxy of cumulative exposure, which is prone to misclassification as to the degree of exposure. No distinction between long-term but low level exposure and short-term but high level exposure was possible.

While employment duration from 1–9 years and ≥20 years indicated an increased risk for lung cancer, no association for employment duration of 10–19 years was observed. The authors speculated that the finding may reflect healthy worker survivor effect (HWE), although the HWE has not been well studied in case-control studies; thus, this interpretation is not justified. Due to the case-control design, the study is also prone to recall bias with respect to exposure assessment. Recall bias can occur in case-control studies, in which cases are asked to recall events from many years ago. It has been repeatedly shown that people with serious illness tend to inaccurately recall events associated with historical hazardous exposures and thus present challenges in the interpretation of the results of case-control studies (30–32). Recall bias tends to be most prominent if the disease is highly significant (such as lung cancer), and the patient has a preconception that the exposure is related to the illness.

In summary, the Taeger study did not have measured exposure data on coal mine dust or confounders, such as crystalline silica, and thus does not provide adequate information to reliably address coal mine exposure and risk of lung cancer. Confounding from carcinogens such as crystalline silica, asbestos, PAH, radon and metals could not be addressed. Employment duration was used as proxy of cumulative exposure, which is prone to misclassification as to the degree of exposure. Recall bias-major limitation in case-control studies. The observed association is unlikely to be directly attributed to coal dust. Assessment of risk is most appropriately based on cohort studies as it avoids inherent biases, most notably recall bias, in case-control studies. The weight of the scientific literature suggests that coal mine dust has little or no effect on lung cancer risk (5).

Tomášková et al. (27) compared two cohorts with respect to total mortality and cause-specific mortality, and lung cancer risk to address whether CWP would accelerate the development of lung cancer. The authors defined a cohort of coal miners with acknowledged coal workers' pneumoconiosis (CWP), and another one without CWP for comparison of the outcomes of interest in the Czech Republic through the period 1992–2013.

The cohort of 6,687 former coal miners who did not have CWP through 2013 yielded a significant decreased mortality risk of lung cancer in comparison to the general population (SMR = 0.83, 95% CI: 0.70–0.98), while the coal miners with CWP had a two-fold higher risk (SMR = 1.70, 95% CI: 1.40-2.04). Furthermore, the authors found that the total mortality of the cohort of 6,687 former coal miners without CWP (SMR = 0.86, 95% CI: 0.82–0.91) was significantly decreased than that of the cohort with CWP (SMR = 1.10, 95% CI: 1.02–1.17), compared to the general population. The mean age at death for coal miners with CWP from diseases of the respiratory system was 70.5 (SD: 10.9), while those without CWP died at age 61.1

To address whether CWP accelerates the development of lung cancer, one needs information on the pathway from (1) coal dust exposure, (2) time to development of CWP, and (3) finally time to development of lung cancer. Using time-to-event data is more reasonable to clarify this question. Unfortunately, the authors used SMR instead, which may explain the contradictory findings between the SMR estimates and age at death between the cohorts. In addition, Tomaskova et al. used maximal permissible exposure as a proxy of exposure for the non-CWP cohort, while the exposure level of the CWP cohort remained unknown.

In summary, the study of Tomaskova et al. did not provide sufficient data to evaluate the risk of coal dust exposure on lung cancer risks and to address whether CWP would accelerate the risk of developing lung cancer. Inherent limitations of the study, including the retrospective design, lack of information on exposure of interest and potential confounding factors, such as silica and smoking habits affect drawing definitive conclusions about risk of lung cancer in this cohort. Further commentary on this study is provided by Yong (33).

### Rats, Monkeys and Exposure to PSLTs

In light of the different reactions of monkeys and rats to inhaled particles, it is wise to address two year inhalation studies of rats exposed to coal dust. In a study of rats and monkeys exposed to coal dust at 2 mg/m^3^ for 7 h/day, 5 days per week for 24 months, rats, but not monkeys, had significant alveolar epithelial hyperplastic, inflammatory, and septal fibrotic responses to the retained particles (34). The response to particles, including alveolar epithelial hyperplasia, inflammation and focal septal fibrosis, was significantly greater in rats than in monkeys. Rats had a significantly greater alveolar epithelial hyperplastic response to particle exposure than monkeys (*p* < 0.001). Rats also had a significantly greater inflammatory response to particles than monkeys (*p* = 0.02). Lung tumors were not demonstrated in either monkeys or rats at exposures of 2 mg/m^3^. The authors concluded that “*if human lungs respond to poorly soluble particles in a manner more like monkey lungs than rat lungs, perhaps the pulmonary response of rats particles may not be predictive of the response in human lungs at concentrations representing high occupational exposures”* (34).

## Discussion

### Is Coal a PSLT and a Suitable Substitute for Carbon Black and TiO_2_?

Substantial compositional differences exist between coal, carbon black and TiO_2_.

Coal contains significant concentrations of crystalline silica (Type I IARC carcinogen).

Coal mining environment often includes exposure to diesel exhaust particles, another IARC Type I Human carcinogen. As a result, considering coal as a poorly soluble particle is not scientifically justified.

### Coal Worker Cohort Mortality Studies

“Using a weight of evidence approach, studies of coal-mine workers, who have been exposed to occupationally relevant levels of dust, do not indicate an increase in lung cancer risk. Classifying all poorly soluble as carcinogenic to humans based on rat inhalation studies in which lung overload leads to chronic inflammation and cancer is not supported by data in humans” [Morfeld et al. (5), p. 12:3, *Particle and Fiber Toxicology*].

## Conclusion

Using a *weight of evidence* approach-considered the preferred method when evaluating disparate studies to assess risk- studies of coal-mine workers do not indicate a consistent increase in lung cancer risk. Slight elevations in SMR cannot lead to a reliable conclusion about an increased risk due to limitations in exposure assessment and control of inherent biases in case-control studies, most notably control of confounding and recall bias. In conclusion, the weight of the scientific literature suggests that coal mine dust does not increase lung cancer risk. And finally, due to substantial compositional differences between coal dust, carbon black and titanium dioxide, coal dust cannot be considered representative of a poorly soluble low toxicity particle.

## Author's Note

Based on a presentation at Particles and Health 2021, an international conference held in London, October 2021 and sponsored by the Institute of Occupational Medicine. Edinburgh, Scotland.

## Author Contributions

All authors listed have made a substantial, direct, and intellectual contribution to the work and approved it for publication.

## Conflict of Interest

RM and MY serve as members of the Scientific Advisory Group of the International Carbon Black Association. MY was employed by MY EpiConsulting.

## Publisher's Note

All claims expressed in this article are solely those of the authors and do not necessarily represent those of their affiliated organizations, or those of the publisher, the editors and the reviewers. Any product that may be evaluated in this article, or claim that may be made by its manufacturer, is not guaranteed or endorsed by the publisher.
